# Digital health in the Americas: advances and challenges in connected health

**DOI:** 10.1136/bmjinnov-2017-000258

**Published:** 2018-05-26

**Authors:** David Novillo-Ortiz, Elsy Maria Dumit, Marcelo D’Agostino, Francisco Becerra-Posada, Edward Talbott Kelley, Joan Torrent-Sellens, Ana Jiménez-Zarco, Francesc Saigí-Rubió

**Affiliations:** 1 Department of Evidence and Intelligence for Action in Health, Pan American Health Organization, Washington, District of Columbia, USA; 2 Department of Service Delivery and Safety, World Health Organization, Geneva, Switzerland; 3 Faculty of Economics and Business, Universitat Oberta de Catalunya, Barcelona, Spain; 4 Faculty of Health Sciences, Universitat Oberta de Catalunya, Barcelona, Spain

**Keywords:** global health, mhealth, telemedicine, universal coverage

## Abstract

In 2005, all WHO Member States pledged to fight for universal health coverage (UHC). The availability of financial, human and technological resources seems to be necessary to develop efficient health policies and also to offer UHC. One of the main challenges facing the health sector comes from the need to innovate efficiently. The intense use of information and communication technologies (ICTs) in the health field evidences a notable improvement in results obtained by institutions, health professionals and patients, principally in developed countries. In the Americas, the relationship between economic development and health innovation is not particularly evident. Data from 19 of 35 countries surveyed in the 2015 Third Global Survey on eHealth for the region of the Americas were analysed. 52.6% of the countries of the Americas have a national policy or strategy for UHC. 57.9% of the countries in the sample indicate that they have a national eHealth policy or strategy, but only 26.3% have an entity that supervises the quality, safety and reliability regulations for mobile health applications. The survey data indicate that high-income and low-income to middle-income countries show higher percentages in relation to the existence of entities that promote innovation. These countries also exceed 60%—compared with 40% and 50% in lower-income countries—in all cases regarding the use of eHealth practices, such as mobile health, remote patient monitoring or telehealth. 100% of low-income countries report offering ICT training to healthcare professionals, compared with 83% of wealthy countries and 81% of middle-income to high-income countries.

## Introduction

The health sector is a priority for governments and supranational institutions. WHO defines *health systems* as those that are working in harmony and are ‘built on having trained and motivated health workers, a well-maintained infrastructure, and a reliable supply of medicines and technologies, backed by adequate funding, strong health plans and evidence-based policies’.^1^ The activities that these institutions and actors develop are aimed at preventing and controlling disease, treating the sick, and conducting research and training in health. This is why the health sector has a direct impact on several of the United Nations’ Sustainable Development Goals.^2^ Although the health sector’s importance is more than evident at the social level, its importance at the economic level, recognised over recent years, should also be noted. According to the World Bank (2017), health spending represented 12.3% of the gross domestic product (GDP) of the countries of the Organization for Economic Cooperation and Development in 2014, and its contribution to economic growth in some countries is between 3.5% and 5.5%.^3^

One of the main challenges facing the health sector comes from the need to innovate efficiently. This is so that, on the supply side, health institutions can perform care processes more quickly, accurately and economically; and on the demand side, patients can receive a more responsive and higher quality service that is better adapted to their needs and circumstances, thus improving their experience throughout the entire process.

The intense use of information and communication technologies (ICTs) in the health field evidences a notable improvement in results obtained by institutions, health professionals and patients.^4^

In the Americas, the relationship between economic development and health innovation is not particularly evident.^5^ Specifically, low-income to middle-income economies (deemed so based on the International Monetary Fund wealth index) allocate a high percentage of their GDP to health expenditures while showing innovation indicators equal to or higher than those of high-income economies such as the USA and Canada.

Given this situation, the present work aims to determine whether a nation’s striving towards the health and well-being of its citizens depends solely on the level of economic development, or whether it also depends on the social actions of its leaders. To do this, we have taken the region of the Americas and its experiences in the development of digital health (or eHealth) as a unit of analysis. Data from the Third Global Survey on eHealth for the region of the Americas seem to show that health and well-being depend on both countries’ economic development and the social actions of its leaders since, regardless of governments’ political leanings (which may be totally opposed as in countries such as Cuba and Honduras), the indicators related to certain health management practices are high. Furthermore, the policies developed in the healthcare arena dub universal coverage, eHealth and technology training for healthcare professionals as key in health policy.

## Methodology

We analysed the data from the Third Global Survey on eHealth from 2015 for the region of the Americas. Of the 35 member countries, data pertaining to 19 (54.9%) were obtained. Analysis was carried out using univariate and bivariate statistical methods. For this reason, the results are mainly presented as a percentage of the total number of members that answered a question or as the absolute number of countries that gave a particular response.

### Global Survey on eHealth

The Third Global Survey on eHealth is an instrument developed by WHO’s Global Observatory for eHealth,^6^ based on the consultation with, and information from, some of the strategic partners of WHO and the Pan American Health Organisation: governments, collaborating centres, professional associations and international organisations. The survey is modified and updated based on exhaustive analysis of information collected from the responses of the Member States. The purpose of WHO’s digital health studies carried out worldwide is to observe and determine the reference points in the adoption and progress of eHealth at the national, regional and global levels. The first survey was conducted in 2005 and its objective was to collect information at the national level in order to establish an analysis of the existing situation. The second survey, conducted in 2009 and based on the first survey, incorporated new questions and included a new approach on the subject of eHealth. The 2015 survey examined digital health in the context of its role in supporting universal health coverage (UHC).^7^ The objectives of this third survey were to measure, at a global level, progress in the development of eHealth and to later compare these results with those obtained in previous studies in order to identify current barriers and explore future trends in terms of digital health.

## Results

The region is characterised by a strong cultural, social, linguistic, demographic and economic diversity that exists among the 35 countries that comprise it. A result of the region’s historical tradition, this situation also influences the way of approaching and managing health. Therefore, for some countries, the level of economic development—or even the political orientation of governments—seems to have no impact on the way health is managed (in particular, the degree of social coverage offered or the level of innovation achieved).

### Social approach to health policy: national coverage and eHealth plans

Health is a matter of great social and economic importance for the countries in the American continent. Yet, the health policies implemented by the different states show great disparity, both in terms of the level of coverage offered and in relation to the implementation of eHealth policies.

The survey data show that 52.6% of the countries of the Americas have a national policy or strategy for UHC. However, this percentage is not consistent across the region, especially when considering income levels; contrary to what might be expected, 100% of low-income countries have a UHC policy, compared with 50% and 54.5% of countries with high and middle-to-high incomes, respectively (table 1).

**Table 1:** Health policy indicators by countries according to their level of income, universal health coverage, and information and communication technology use

	All countries (%)	High-income countries (%)	Middle-income to high-income countries (%)	Low-income to middle-income countries (%)
Universal health coverage	52.6	50.0	54.5	100
eHealth strategy	57.9	83.3	36.4	100
Electronic health records	52.6	50.0	54.5	50.0
Entity that oversees eHealth	26.3	33.4	9.1	100
Entity incentive on research, development and innovation	63.2	83.3	45.5	100

A similar situation was observed in the development of eHealth practices. The level of establishment of digital health and the existence of a regulatory system for this type of practice are not precisely linked to a country’s level of economic development. In general terms, 57.9% of the countries in the sample indicate that they have a national eHealth policy or strategy, 52.6% report having a national electronic health record system, but only 26.3% have an entity that supervises the quality, safety and reliability regulations for mobile health applications (table 1).

As in the previous case, these percentages are higher for countries with high or low incomes. The countries have no significant differences in the existence of an electronic health record. However, in eHealth and the availability of a supervisory entity for eHealth, the differences are notable, with these entities existing in 100% of low-income to middle-income countries.

## Discussion

### Health: a multifaceted reality

Beyond agreeing on a definition for health^8^—a fundamental yet controversial issue—it is imperative to establish the way in which governments and organisations have to design and carry out policies for their management.

In 2005, all WHO Member States pledged to fight for UHC; this represented a collective expression of the belief that all people should have access to the health services they need without risk of financial ruin or impoverishment.^9^

From a social point of view, working towards UHC is a powerful mechanism to achieve better health and well-being outcomes, as well as to promote human development. In fact, WHO’s declaration of its intentions for UHC automatically deems health and social well-being a universal right. Nonetheless, any action involves the use of resources, so along with the social dimension, health also has an economic dimension that influences the way in which each country orients its health policies.^10^

Accordingly, achieving improvements in the relationship between the quantity and quality of life gained and the resources used to obtain such gains requires adequate health policies, but, above all, it is about making cost-effective clinical decisions.^11^ The efficiency of a health system depends essentially on health institutions and organisations, as well as healthcare professionals, having the necessary information, training and incentives. They are the ones who make most of the decisions regarding prevention, diagnosis, treatment and rehabilitation. For that reason, the availability of financial, human and technological resources seems to be necessary to develop efficient health policies and also to offer UHC.^12^

As Niembro *et al* 13 point out, the aforementioned idea that resource availability is essential for UHC shows a direct relationship between a country’s level of economic development and its health policies. Yet, the results of the Third Global Survey on eHealth call into question some of the previous statements. For instance, contrary to expectations, some countries with scarce economic resources allocate abundant technological, human and economic resources to health management, offering UHC and also investing in highly innovative, competitive and efficient healthcare management.

### Economic development in the Americas: the health sector

One of the main characteristics of the region of the Americas is the different levels of economic development of its countries. According to data from the International Monetary Fund,^14^ 82.2% of the countries in the region can be classified as middle-income to high-income economies, whose per capita income in US dollars is between 2500 and 25 000 per year. Only 5.3% are considered high-income countries while 11.4% are classified as low-income to middle-income countries (below US$2500 per year per capita).

For all the countries in the region, health is a matter of priority. However, as the World Bank indicates,^15^ the importance of health expenditure for the Americas, as a percentage of total GDP, is a true reflection of the level of economic development of the countries (total health expenditure is the sum of public and private health expenditure, covering the provision of preventative and curative health services, family planning activities, nutrition activities and emergency healthcare assistance, but not including water supply or sanitary services). Consequently, high-income countries show health expenditures from 11.5% in Canada to 17.1% in the USA, whereas in lower-income countries, health expenditure does not exceed 6%–9% of GDP; for example, Nicaragua and the Dominican Republic have health expenditures of 3.6% and 5.6%, respectively. However, some low-income to middle-income countries do not display this pattern of behaviour; countries such as Haiti, Honduras and Cuba show very high expenditure given their level of economic development.

In the specific case of Haiti, World Bank data show how the percentage of expenditure has decreased from 10.41% in 2011 to 7.5% in 2015. Meanwhile, the economies of Honduras and Cuba show health expenditures reaching 8.72% and 10.40% of GDP, respectively.

This strong investment is manifested in the development and implementation of a health policy oriented towards social well-being, which competes with the most developed economies in terms of innovation.

### Innovation indicators: training and innovative programmes in eHealth

Undoubtedly, innovation is one of the main sources of productivity and efficiency in the provision of health services in the context of the knowledge society. As Hall and Rosenberg^16^ indicate, the main source of innovation is the intentional application of knowledge and technology to develop new or better services, processes and organisational changes. Thus, the main innovation in the field of health comes from the intensive use of ICT, through the development of eHealth programmes and the constant training in technology that health personnel receive.

In the Americas, we can appreciate important differences between the countries both in the disposition of an entity that offers incentives and orientation for innovation, research and evaluation and in the use of advanced practices in eHealth, or the training of health professionals in technology and social networks.

As previously noted, the survey data indicate that high-income and low-income to middle-income countries show higher percentages in relation to the existence of entities that promote innovation (table 1). Likewise, these countries also exceed 60%—compared with 40% and 50% in lower-income countries—in all cases regarding the use of eHealth practices, such as mobile health, remote patient monitoring or telehealth.

Finally, with respect to training of healthcare professionals in the use of ICT, the percentages reported by the low-income countries are higher than those shown by the high-income and middle-income to high-income countries. One hundred per cent of low-income countries report offering ICT training to healthcare professionals, compared with 83% of wealthy countries and 81% of middle-income to high-income countries. It should be noted, however, that regardless of the economic level of the country, training offered to professionals in the use of social networks is relatively low, not exceeding 18%.

## Conclusions

UHC is a citizen’s right, an instrument of economic efficiency, and a mechanism of equality and social well-being. The public policy of economic growth and social welfare should facilitate access to and use of efficient, effective and equitable health services. The new economic and social context posited by the advent of the knowledge society places people at the centre of the competitive and social well-being scenario. In this new context, having a population with an optimal state of health is a fundamental requirement.

The need for resources to carry out public policies that favour UHC, as well as an innovative and efficient health system, leads one to believe that the level of economic development of the country is a determinant for this. Nevertheless, reality allows us to observe that, just as in certain economies where the level of development is low, the effort made—both by public institutions and by organisations and health professionals—is high.

The results from the Third Global Survey on eHealth for the region of the Americas primarily show that the use of ICTs has intensified, and that the level of income, as well as countries’ health expenditure, are not observed as a determining variable in the implementation of eHealth programmes. It would be up to the countries to bet on the benefits of the use of eHealth, which would favour its implementation. This is the case of economies considered poor ones, which allocate high percentages of their GDP towards health expenditure. In that vein, these countries strive to offer UHC, to develop advanced practices in the field of eHealth, to train their health professionals in the field of ICTs and to have advanced legislation in the field of digital health. This could explain how Cuba, a country qualified as low-middle income and with low healthcare expenditure, has a public, universal and free healthcare system. To this system an increasing development of the services infrastructure and human capital is added, along with a significant number of eHealth programmes, making Cuba one of the nations with the best health indicators in the world.^5^
